# Sodium chloride effect on the aggregation behaviour of rhamnolipids and their antifungal activity

**DOI:** 10.1038/s41598-017-13424-x

**Published:** 2017-10-10

**Authors:** Ana I. Rodrigues, Eduardo J. Gudiña, José A. Teixeira, Lígia R. Rodrigues

**Affiliations:** CEB - Centre of Biological Engineering, University of Minho, 4710-057 Braga, Portugal

## Abstract

In this work, the antifungal activity of rhamnolipids produced by *Pseudomonas aeruginosa* #112 was evaluated against *Aspergillus niger* MUM 92.13 and *Aspergillus carbonarius* MUM 05.18. It was demonstrated that the di-rhamnolipid congeners were responsible for the antifungal activity exhibited by the crude rhamnolipid mixture, whereas mono-rhamnolipids showed a weak inhibitory activity. Furthermore, in the presence of NaCl (from 375 mM to 875 mM), the antifungal activity of the crude rhamnolipid mixture and the purified di-rhamnolipids was considerably increased. Dynamic Light Scattering studies showed that the size of the structures formed by the rhamnolipids increased as the NaCl concentration increased, being this effect more pronounced in the case of di-rhamnolipids. These results were confirmed by Confocal Scanning Laser Microscopy, which revealed the formation of giant vesicle-like structures (in the µm range) by self-assembling of the crude rhamnolipid mixture in the presence of 875 mM NaCl. In the case of the purified mono- and di-rhamnolipids, spherical structures (also in the µm range) were observed at the same conditions. The results herein obtained demonstrated a direct relationship between the rhamnolipids antifungal activity and their aggregation behaviour, opening the possibility to improve their biological activities for application in different fields.

## Introduction

Moulds play an important role in the spoilage of agriculture products, especially during storage. Fungal contaminations can be disastrous to crops despite the preventive measures adopted for their control. Management of fungal contaminations is generally a difficult challenge, since once initiated, epidemics are very difficult to contain^1,2^. It is estimated that 5 to 10% of the World’s food production is lost due to fungal contamination, which causes high economic losses and several health risks due to the toxicity and pathogenic nature of some species^2,3^. The extensive use of chemical fungicides to control plant diseases has a negative impact in the ecological balance of microorganisms inhabiting soil, due to the development of resistant strains of pathogens, groundwater contamination, and health risks to humans^1,4^. Species belonging to the genus *Aspergillus* are among the most common fungal contaminants, being some of them pathogenic for plants and animals^5^. Among them, *Aspergillus niger* and *Aspergillus carbonarius* are capable of growing in a wide range of organic substrates, causing deterioration of stored food material. Furthermore, *A. carbonarius* is one of the most important opportunistic pathogens of grapes^6,7^.

The development of novel, more effective, safe and environmental friendly antifungal agents as an alternative to the chemical fungicides used for combating a variety of crop diseases is currently an environmental challenge^2,4^. Several biosurfactants have been reported as effective biocontrol agents; however, only few reports describe rhamnolipids (RLs) for this application. RLs exhibit antagonistic activity against some fungi^8–12^ and have been demonstrated to inhibit pathogenic fungi resistant to commercial chemical pesticides^13^. Moreover, RLs stimulate plant immunity, which can be considered as an alternative strategy to reduce the infections caused by plant pathogens^2,12^.

RLs are glycolipid biosurfactants produced mainly by *Pseudomonas* and *Burkholderia* species, being *Pseudomonas aeruginosa* the main producer^14,15^. The structure of RLs comprises one (mono-RLs) or two (di-RLs) rhamnose molecules linked to one or two β-hydroxy fatty acids which can differ in the chain length (8–24 carbons) and the degree of saturation (0–2 double bonds)^13^. RLs are usually produced as a mixture of different congeners, and the ratio between mono-RLs and di-RLs depends on the bacterial strain, the culture medium composition and the operational conditions^16,17^. RLs can be synthesized using renewable low-cost substrates, such as plant oil and grain starch^18^, wastes from sunflower oil^19^, sugarcane molasses^20,21^, corn steep liquor (CSL)^21^ and olive oil mill wastewater^16^, which could contribute to reduce their production costs. Due to their excellent surface activity, together with their environmental friendly nature, they can be used as wetting, sticker or dispersal agents for application in fungicides or pesticides. Indeed, RLs are already applied in the formulation of the commercial biofungicide Zonix^TM^ (NOP Supply LLC, USA).

The aim of this work was to evaluate the antifungal activity of RLs produced by *P. aeruginosa* #112 against *A. niger* and *A. carbonarius*. A low-cost culture medium, containing sugarcane molasses and CSL, was used for rhamnolipid production. Furthermore, it was studied which congeners (mono-RLs or di-RLs) were responsible for the antifungal activity observed. Finally, the relationship between the antifungal activity and the aggregation behaviour of these RLs was studied.

## Results and Discussion

### Purification of mono-RL and di-RL congeners produced by *P. aeruginosa* #112

*P. aeruginosa* #112 was previously reported to produce eight different RL congeners using the culture medium CSLM. Among them, the most abundant were the mono-RL Rha-C_10_-C_10_ and the di-RL Rha-Rha-C_10_-C_10_, although lower amounts of other congeners with acyl chains containing 8 or 12 carbons were also identified^21^. The mono-RL and di-RL congeners present in the crude RL mixture were separated through silica gel column chromatography. Two different fractions were obtained and these were analyzed through TLC in order to confirm the presence of mono-RLs or di-RLs. As expected, each fraction exhibited a single spot, which R_f_ values were approximately 0.70 and 0.47 for the mono-RL and di-RL samples, respectively. These results are in accordance with previous reports^13,22,23^. After the separation process, 500 mg of the crude RL mixture yielded 51.7 ± 7.6 mg of mono-RLs and 149.0 ± 48.9 mg of di-RLs. According to the results obtained, only 40% of the crude RL sample corresponds to RLs, thus it can be assumed that the remaining 60% were impurities.

### Antifungal activity of the RL mixture produced by *P. aeruginosa* #112

In a first approach, the antifungal activity of the RLs produced by *P. aeruginosa* #112 against the fungi *A. niger* MUM 92.13 and *A. carbonarius* MUM 05.18 was evaluated using the cell-free supernatant obtained at the end of the fermentation performed in the culture medium CSLM, which contains about 3 g of RLs per litre^21^. The cell-free supernatant completely inhibited the growth of *A. carbonarius* MUM 05.18, whereas in the case of *A. niger* MUM 92.13, 75.5 ± 2.7% of growth inhibition was achieved. These results are in good agreement with previous studies that reported the antagonistic activity of RLs against other genera of fungi, including *Botrytis*, *Colletotrichum*, *Cylindrocarpon*, *Fusarium*, *Magnaporthe*, *Mucor*, *Phytophthora* and *Macrophomina* ^9–11,13^. Although the mechanisms responsible for this antifungal activity are not well established, it is assumed that low molecular weight biosurfactants like RLs interact with the lipid constituents of biological membranes, disturbing their integrity and permeability by inducing the formation of pores and ion channels^2,24^.

Subsequently, the antifungal activity was studied using the partially purified crude RL mixture at different concentrations (0.375–12 g/L). Surprisingly, the growth inhibition percentages obtained for a RL concentration of 3 g/L were considerably lower (28.0 ± 8.6% for *A. niger* MUM 92.13 and 22.6 ± 1.2% for *A. carbonarius* MUM 05.18) when compared with those achieved with the cell-free supernatant. Even using a crude RL concentration of 12 g/L, low growth inhibition percentages (between 28 and 30%) were found. Similarly to the results herein obtained, Sha and co-workers^13^ reported that the cell-free supernatant containing RLs exhibited a higher antifungal activity against several plant pathogens when compared with the purified mono-RL and di-RL congeners. In this case, the authors suggested that this could be due to the presence of both types of RLs in the cell-free supernatant. Haba and co-workers^25^ also reported that a purified mixture of mono-RL and di-RL congeners exhibited a weak inhibitory activity against several yeast and fungal strains (including *A. niger*), although it showed a considerable antimicrobial activity against several Gram-positive and Gram-negative bacteria.

Due to the discrepancy observed between the antifungal activities exhibited by the cell-free supernatant and the crude RL mixture, further studies were performed to establish if other compounds (instead the RLs) present in the culture medium CSLM or in the cell-free supernatant were responsible for the antifungal activity. These studies were performed in the same way that those previously described, but replacing the cell-free supernatant containing RLs by the culture medium CSLM or by the cell-free supernatant after removing the RLs. In none of these studies was observed antifungal activity, suggesting that it must be due to the RLs. It has been previously reported that the biological activity of RLs is related to their aggregation behaviour, which is affected by the pH and the presence of electrolytes^26,27^. In order to validate this hypothesis, the antifungal activity of the crude RL mixture was studied in the presence of different NaCl concentrations. Control assays were performed using the culture medium MEA supplemented with the same NaCl concentrations tested (250–1000 mM), and it was found that they did not inhibit the fungal growth. On the contrary, in the case of *A. niger* MUM 92.13, the addition of NaCl into the culture medium at concentrations between 750 and 1000 mM enhanced the fungal growth by 8.9 and 19.8%, respectively.

It was found that, for both fungi, the addition of NaCl to the culture medium increased the antifungal activity of the crude RL mixture. The lowest NaCl concentrations that resulted in a complete growth inhibition in combination with the crude RL mixture (3 g/L) were 875 mM for *A. niger* MUM 92.13 (Table 1) and 375 mM for *A. carbonarius* MUM 05.18 (Table 2). For the other RL concentrations tested (0.375, 0.75 and 1.5 g/L), the addition of NaCl (875 mM for *A. niger* MUM 92.13 and 375 mM for *A. carbonarius* MUM 05.18) to the culture medium also increased the antifungal activity when compared with the assays performed without NaCl (Tables 1 and 2). The explanation for this behaviour could be that the combination of NaCl with the crude RL mixture allowed the formation of structures similar to those originally present in the cell-free supernatant, which confer the antifungal activity to the RLs. This organization must be lost during the recovery of the RLs, which resulted in a considerable reduction of their antifungal activity.

**Table 1:** Growth inhibition percentages obtained for *Aspergillus niger* MUM 92.13 with the crude rhamnolipid mixture produced by *Pseudomonas aeruginosa* #112. The assays were performed at different concentrations of the crude rhamnolipid mixture in the presence of different NaCl concentrations, at 25 °C for 5 days. The results represent the average of three independent experiments ± standard deviation. [RL]: concentration of crude rhamnolipid mixture. NT: not tested.

[RL] (g/L)	[NaCl] (mM)				
	0	500	750	875	1000
	Growth inhibition (%)				
0.375	17.1 ± 6.5	NT	NT	43.3 ± 1.2	NT
0.75	8.1 ± 3.7	NT	NT	53.2 ± 0.0	NT
1.5	13.0 ± 3.7	NT	NT	51.8 ± 1.2	NT
3.0	28.0 ± 8.6	29.1 ± 1.2	73.0 ± 1.1	100.0 ± 0.0	100.0 ± 0.0

**Table 2:** Growth inhibition percentages obtained for *Aspergillus carbonarius* MUM 05.18 with the crude rhamnolipid mixture produced by *Pseudomonas aeruginosa* #112. The assays were performed at different concentrations of the crude rhamnolipid mixture in the presence of different NaCl concentrations, at 25 °C for 5 days. The results represent the average of three independent experiments ± standard deviation. [RL]: concentration of crude rhamnolipid mixture. NT: not tested.

[RL] (g/L)	[NaCl] (mM)			
	0	250	375	500
	Growth inhibition (%)			
0.375	24.0 ± 2.1	NT	50.9 ± 6.5	NT
0.75	26.7 ± 10.3	NT	47.2 ± 3.8	NT
1.5	29.5 ± 8.3	NT	58.5 ± 3.8	NT
3.0	22.6 ± 1.2	78.0 ± 1.1	100.0 ± 0.0	100.0 ± 0.0

### Antifungal activity of mono-RL and di-RL congeners

The antifungal activity of the purified mono-RL and di-RL congeners against *A. niger* MUM 92.13 and *A. carbonarius* MUM 05.18 was studied at different concentrations (0.75–1.5 g/L for mono-RLs and 0.05–1.5 g/L for di-RLs), with and without NaCl, in order to evaluate their individual contribution. As it can be seen from the results obtained (Tables 3 and 4), the di-RL congeners were responsible for the antifungal activity observed with the crude RL mixture, whereas the mono-RLs exhibited a weak inhibitory activity even at the highest concentration tested (1.5 g/L). As in the case of the crude RL mixture, the antifungal activity exhibited by the di-RL congeners was enhanced by the addition of NaCl. The purified di-RLs completely inhibited the growth of *A. niger* MUM 92.13 and *A. carbonarius* MUM 05.18 at concentrations of 0.2 and 0.75 g/L, respectively, but only when supplemented with the optimum NaCl concentrations (Tables 3 and 4).

**Table 3:** Growth inhibition percentages obtained for *Aspergillus niger* MUM 92.13 with the purified mono-rhamnolipid and di-rhamnolipid congeners. The assays were performed at different concentrations of the purified rhamnolipid congeners with and without NaCl, at 25 °C for 5 days. The results represent the average of three independent experiments ± standard deviation. [RL]: concentration of mono-rhamnolipid or di-rhamnolipid congeners. NT: not tested.

[RL] (g/L)	Mono-RL	Mono-RL + 875 mM NaCl	Di-RL	Di-RL + 875 mM NaCl
	Growth inhibition (%)			
0.05	NT	NT	NT	52.4 ± 1.2
0.1	NT	NT	NT	61.9 ± 1.2
0.2	NT	NT	NT	100.0 ± 0.0
0.375	NT	NT	41.0 ± 1.5	100.0 ± 0.0
0.75	NT	21.1 ± 2.4	NT	100.0 ± 0.0
1.5	46.2 ± 0.0	41.8 ± 1.4	40.0 ± 7.5	NT

**Table 4:** Growth inhibition percentages obtained for *Aspergillus carbonarius* MUM 05.18 with the purified mono-rhamnolipid and di-rhamnolipid congeners. The assays were performed at different concentrations of the purified rhamnolipid congeners with and without NaCl, at 25 °C for 5 days. The results represent the average of three independent experiments ± standard deviation. [RL]: concentration of mono-rhamnolipid or di-rhamnolipid congeners. NT: not tested.

[RL] (g/L)	Mono-RL	Mono-RL + 375 mM NaCl	Di-RL	Di-RL + 375 mM NaCl
	Growth inhibition (%)			
0.375	NT	NT	34.9 ± 1.3	72.6 ± 1.3
0.5	NT	NT	NT	80.7 ± 1.2
0.6	NT	NT	NT	73.8 ± 1.2
0.75	NT	25.2 ± 4.4	NT	100.0 ± 0.0
1.5	30.2 ± 5.3	26.4 ± 2.7	33.1 ± 3.2	NT

Previous studies suggested that the biological activity associated to RLs is due to the di-RL congeners. Sha and co-workers^13^ reported that di-RLs exhibit a higher antifungal activity against different plant pathogens when compared with mono-RLs, which exhibit only a weak inhibitory activity. Likewise, it was reported that mono-RLs inhibit the growth of *A. niger* only at very high concentrations (68 g/L)^28^. One of the mechanisms proposed for the biological activities of biosurfactants is their interaction with the phospholipid bilayer, which results in a detergent-like effect that disrupts the plasma membrane^29^. This interaction may be influenced by the composition of the hydrophilic head and hydrophobic tails of biosurfactants. It has been proposed that the large polar head group of di-RLs confers them an inverted-cone shape, which induces a positive curvature to the membranes and might be responsible for their disrupting effect^27,30^. However, contrary to our results, other authors reported that mono-RLs exhibit a higher antimicrobial activity against Gram-positive and Gram-negative bacteria when compared with di-RLs^31^. Christova and co-workers^32^ also reported that the mono-RL congener Rha-C_10_-C_10_ showed a higher cytotoxic activity against different human cancer cell lines when compared with the di-RL congener Rha-Rha-C_10_-C_10_.

### Effect of NaCl on the surface activity and the aggregation behaviour of RLs

The purified mono-RLs and di-RLs dissolved in demineralised water (500 mg/L) reduced the surface tension up to 25.9 ± 0.2 mN/m (*cmc* 50 mg/L) and 33.5 ± 0.3 mN/m (*cmc* 15 mg/L), respectively. The higher surface activity of mono-RLs when compared with di-RLs is attributed to their less hydrophilic character, which has been extensively reported in the literature^21,31,33^. RLs have one or two hydrophobic chains, whereas their hydrophilic moieties are the rhamnosyl and the carboxylic groups. Mono-RLs have a single rhamnosyl group, whereas di-RLs have two. The rhamnosyl groups endow hydrophilicity to RLs, while the carboxylic groups carry out the functional control of their amphipathic properties, which are strongly affected by the pH and the presence of electrolytes^33^. According to the results herein obtained, higher *cmc* values have been reported for mono-RLs (0.050 mM, ≈25 mg/L) when compared with di-RLs (0.010 mM, ≈6.5 mg/L) by other authors^24,34^.

Subsequently, it was studied the effect of NaCl on the surface tension and the *cmc* values. In the case of mono-RLs, the addition of NaCl (375 mM and 875 mM) did not have effect on the surface tension values, but resulted in a considerable decrease of the *cmc* (25 mg/L) in both cases. In the case of di-RLs, a slight decrease in the surface tension was observed (31.7 ± 0.1 mN/m) with the highest NaCl concentration tested (875 mM), but the *cmc* value remained constant. The *pKa* values reported for mono-RLs and di-RLs are 5.9 and 5.6, respectively^24,34^. The pH of the RL solutions herein used was not adjusted, and it was around 6.2. In these conditions, in the absence of an electrolyte, the majority of the carboxylic groups of RLs are dissociated to form carboxylate ions. As a result, the liquid surface exhibits a net negative charge, resulting in strong repulsive electrostatic forces between the RL molecules. In the presence of NaCl, this negative charge is shielded by the Na^+^ ions, causing the formation of a close-packed monolayer, which should result in a decrease in the *cmc* and the surface tension values^24,33,34^. This compaction must be higher in the case of mono-RLs when compared with di-RLs, due to the larger di-rhamnose head group, which impose higher packing constraints^33,35^. Although in this case the surface tension values were not affected by the addition of NaCl, this could explain why the *cmc* value of mono-RLs was reduced in the presence of NaCl, whereas in the case of di-RLs it was not affected. On the contrary, other authors proposed that RLs exhibit a weakly ionic nature, even at high pH values, which means that their surface tension and *cmc* values are not affected by the presence of electrolytes^35,36^.

Biosurfactants can self-assemble into a wide variety of morphologically different structures, including micelles, vesicles, multilamellar vesicles, lamellar structures or non-organized multilayers, among others^37^. It has been reported that the different aggregation behaviours are related with the biological activities exhibited by biosurfactants^24,38^. In order to establish a relationship between the organization of RLs and the antifungal activities observed, the micellar size distribution of RLs in aqueous solution was studied at different NaCl concentrations through DLS, using the crude RL mixture, as well as the purified mono-RL and di-RL fractions. The RL concentrations used to perform these assays were selected according to the quality of the measurements (determined by the correlation function and the polydispersity index (PDI) value).

According to the results obtained (Table 5), the purified mono-RLs and di-RLs formed micelles of similar size (130–140 nm) when dissolved in ultrapure water, whereas the crude RL mixture formed micelles around 300 nm. The size of the structures formed by the RLs increased in all the cases as the NaCl concentration increased. This increase was higher for the purified mono-RLs when compared with the crude RL mixture. However, the most significant increase was observed for the di-RL congeners; in this case it was not possible to measure the size of the structures formed for any of the NaCl concentrations tested, as they were out of the range of the equipment (10 µm).

**Table 5:** Effect of NaCl on the micellar size distribution of the crude rhamnolipid mixture and the mono-rhamnolipid and di-rhamnolipid congeners determined by DLS analysis. The concentrations of the crude rhamnolipid mixture, mono-rhamnolipids and di-rhamnolipids were 1.5, 1.0 and 0.5 g/L, respectively. The results represent the average of 10 measurements ± standard deviation. ND: not determined.

	[NaCl] (mM)	Size (nm)	PDI
Crude RL mixture	0	302.8 ± 7.4	0.549 ± 0.009
	375	456.6 ± 42.2	0.596 ± 0.106
	875	2343 ± 154.1	0.753 ± 0.190
Mono-RL	0	140.3 ± 2.0	0.263 ± 0.006
	375	2212 ± 444.1	0.890 ± 0.107
	875	4674 ± 359.8	1.000 ± 0.000
Di-RL	0	133.1 ± 4.9	0.373 ± 0.042
	375	> 10000	ND
	875	> 10000	ND

**Figure 1:**
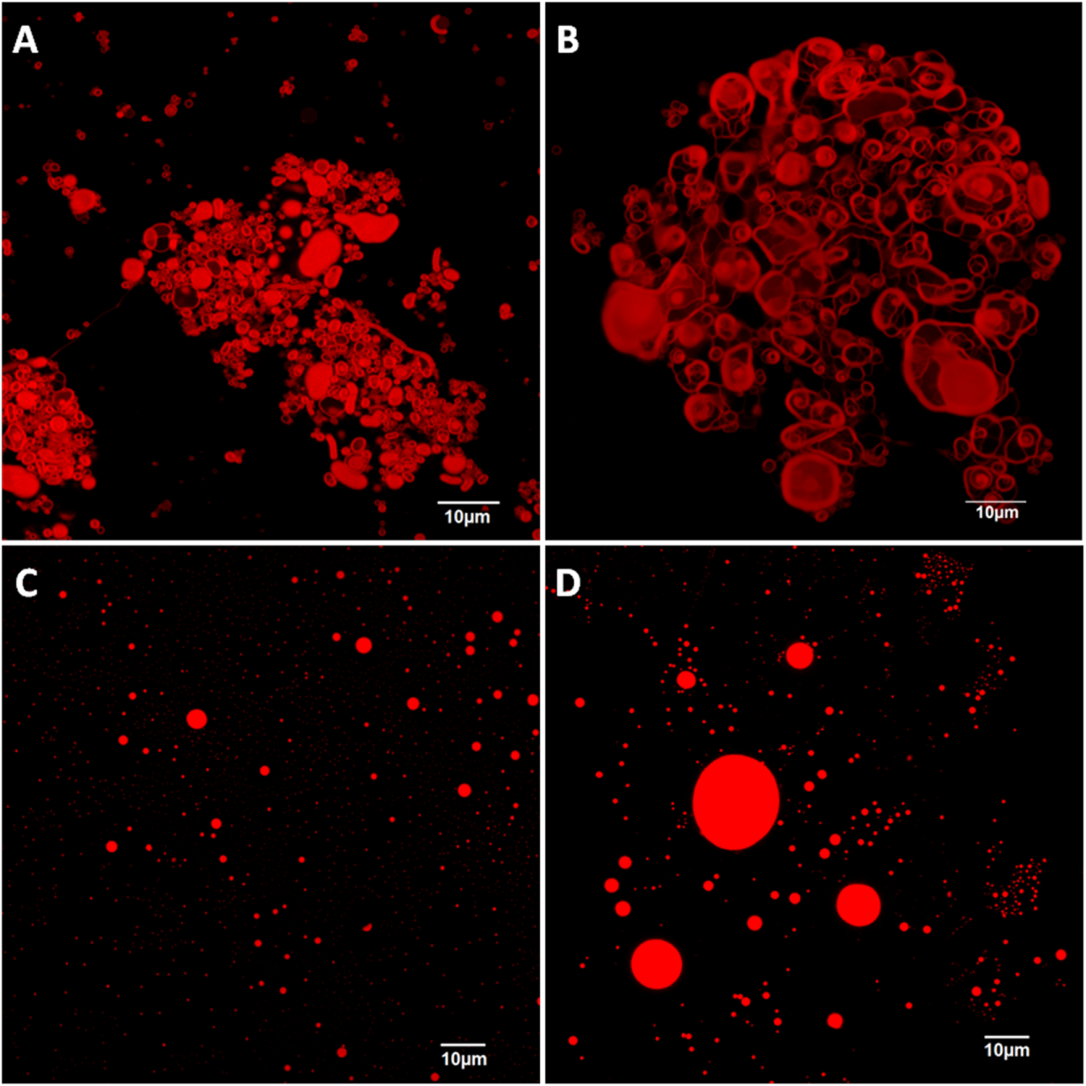
Confocal scanning laser microscopy images showing the self-assembled structures formed by the crude rhamnolipid mixture (**A**,**B**), mono-rhamnolipids (**C**) and di-rhamnolipids (**D**) in the presence of 875 mM NaCl. Nile Red was used as fluorescence probe.

The organization of the crude RL mixture and the mono-RL and di-RL congeners in aqueous solution was analyzed through CSLM (Fig. 1). The images revealed the formation of giant vesicle-like structures (in the µm range) by self-assembling of the crude RL mixture in the presence of 875 mM NaCl. In the case of the purified mono-RLs and di-RLs, spherical structures were observed at the same conditions. The size of the structures formed was in accordance with the results predicted by the DLS measurements. The images also confirmed the heterogeneity of the samples as predicted by the high PDI values (Table 5). None of these structures was observed in the absence of NaCl (*data not shown*). Consequently, it can be suggested that the changes observed in the aggregation behaviour of RLs in the presence of NaCl are responsible for the increase in their antifungal activity against *A. niger* MUM 92.13 and *A. carbonarius* MUM 05.18.

It has been previously reported that the addition of divalent cations to anionic surfactant solutions promoted their micellar growth and a transition from micellar to lamellar structures^36,38,39^. In the case of RLs, the NaCl concentration exhibits a significant effect on the size of the RL aggregates, and it has been reported the formation of spherical micelles, lamellar vesicles, bilayers and rod shaped micelles as a function of the NaCl concentration^26,33^, as well as the transition from micellar to lamellar structures as a result of the addition of electrolytes^35^. In the case of di-RLs, the formation of non-micellar aggregates and lamellar vesicles (with sizes higher tan 1500 nm) in the presence of 150 mM NaCl has been reported by other authors, but only at high di-RL concentrations (2.5 mM, ≈1.6 g/L), whereas at lower di-RL concentrations (1 mM, ≈0.65 g/L), micelles (43–66 nm) and non-micellar aggregates (350–550 nm) coexist in similar proportions^24^. On the contrary, in the case of mono-RLs, even at high concentrations (up to 3 mM, ≈1.5 g/L), the formation of aggregates with a lower average size (200–210 nm) has been reported in the presence of 100 mM NaCl^34^. Micelle-to-vesicle transition was only observed at a very high mono-RL concentration (50–100 mM, ≈25–50 g/L)^35^. In our case, the size of the structures formed by the crude RL mixture and the purified mono-RLs and di-RLs was higher when compared with those reported by other authors. However, the formation of vesicle-like structures was only observed for the crude RL mixture, but not for the purified mono-RLs and di-RLs.

## Conclusions

RLs produced by *P. aeruginosa* #112 in the culture medium CSLM exhibited antifungal activity against *A. niger* and *A. carbonarius* strains, being a promising alternative to the chemical fungicides commonly used. It was demonstrated that the di-RL congeners are responsible for the antifungal activity observed, whereas the mono-RLs exhibited only a weak inhibitory activity. It was also observed that the antifungal activity is lost during the recovery of the RLs from the culture medium, and it was restored by the addition of NaCl, which also altered their aggregation behaviour. Consequently, a relationship between the antifungal activity of RLs and their aggregation behaviour was observed, which can be useful for the development of more effective antifungal agents with better and improved properties.

## Methods

### Microorganisms and culture conditions

The bacterial strain *P. aeruginosa* #112 was used for RLs production. This strain, isolated from a crude oil sample obtained from a Brazilian oil field, was previously reported as a promising RL producer^21^. It was stored at −80 °C in Luria Bertani (LB) medium supplemented with 20% (v/v) of glycerol. The composition of LB medium was (g/L): NaCl 10.0; tryptone 10.0; yeast extract 5.0; pH 7.0. The fungal strains *A. niger* MUM 92.13 and *A. carbonarius* MUM 05.18 were obtained from the culture collection of Micoteca of Universidade do Minho (MUM, Portugal). They were maintained at −80 °C in sterile demineralised water supplemented with 20% (v/v) of glycerol. Whenever required, frozen stocks of the fungal strains were streaked on Malt Extract Agar (MEA) plates and incubated at 25 °C for 7 days. The agar plates were stored at 4 °C no longer than 3 weeks. The composition of MEA medium (Blakeslee’s formula) was (g/L): malt extract 20.0; glucose 20.0; peptone 1.0; agar 20.0; pH 5.5.

### Agro-industrial residues

Sugarcane molasses and CSL were kindly provided by RAR: Refinarias de Açúcar Reunidas, S.A. (Portugal) and COPAM: Companhia Portuguesa de Amidos, S.A. (Portugal), respectively. The composition of these residues was reported in our previous work^21^.

### RLs production and recovery

The production of RLs by *P. aeruginosa* #112 was performed in 1000 mL flasks containing 400 mL of the culture medium CSLM^21^ (sugarcane molasses (10% (w/v)); CSL (10% (v/v)); pH 7.0). Each flask was inoculated with 1% (v/v) of a pre-culture of *P. aeruginosa* #112 grown for 24 h in LB medium at 37 °C and 180 rpm. Subsequently, the flasks were incubated at the same conditions for 120 h. RLs production was monitored along the fermentation by measuring the surface tension of the cell-free supernatants obtained after centrifuging (10000 × g, 20 min, 20 °C) the samples recovered at different time points. At the end of the fermentation, the cultures were centrifuged (10000 × g, 20 min, 20 °C), and the RLs produced were recovered from the cell-free supernatants by adsorption chromatography using the polystyrene resin Amberlite XAD-2 (Sigma-Aldrich Co., USA) as previously described by Gudiña and co-workers^21^. RLs production was determined as the dry weight of the freeze-dried product (crude RL mixture).

### Purification of mono-RLs and di-RLs

The mono-RL and di-RL congeners present in the crude RL mixture were separated and purified through silica gel column chromatography. A 200 mL glass column was filled with silica gel 60 (particle size 63–200 µm, mesh size 70–230 (Sigma-Aldrich Co., USA)) and equilibrated with 100 mL of chloroform at a constant flow rate of 1 mL/min. 500 mg of the freeze-dried crude RL mixture were dissolved in 50 mL of chloroform and loaded onto the column. Subsequently, the column was washed with 100 mL of chloroform to remove the non-adsorbed compounds (i.e. neutral lipids). After that, the mono-RL and di-RL congeners were separated by elution with chloroform:methanol mixtures with increasing polarity: 180:5 (v/v) for mono-RLs and 180:20 (v/v) for di-RLs. All the steps were performed at the same flow rate (1 mL/min). Finally, the solvents were evaporated and the purified mono-RL and di-RL fractions were dissolved in a minimal amount of demineralised water and freeze-dried. The products obtained were weighed and stored at −20 °C for further studies.

The purification process was monitored through thin layer chromatography (TLC). Samples of the different fractions recovered were spotted onto silica gel TLC plates (DC-Fertigfolien ALUGRAM^R^ SIL G UV_254_, Macherey-Nagel GmbH & Co., Germany) which were developed using a solvent system consisting of chloroform:methanol:water (65:25:4, v/v/v). The isolated compounds on the TLC plate were located by spraying it with a solution containing orcinol (0.19% (w/v)) in 53% (w/v) sulphuric acid, followed by incubation at 105 °C until the plots became visible. A sample of the crude RL mixture (containing mono-RLs and di-RLs) was used as reference.

### Surface-activity determination

The surface tension measurements were performed at room temperature (25 °C) according to the Ring method as described elsewhere^40^ using a KRÜSS K20 Tensiometer (KRÜSS GmbH, Germany) equipped with a 1.9 cm du Noüy platinum ring. All the measurements were performed at least in triplicate. The critical micelle concentrations (*cmcs*) of the purified mono-RL and di-RL fractions were calculated as described by Gudiña and co-workers^40^, using the freeze-dried samples dissolved in demineralised water at different concentrations. All the measurements were performed in triplicate.

### Antifungal activity assays

*A. niger* MUM 92.13 and *A. carbonarius* MUM 05.18 were cultured in MEA plates at 25 °C for 7 days. To prepare the spore suspensions, the fungus surface was washed with 1 mL of sterile demineralised water, which was subsequently transferred to a sterile tube. The spore suspensions were diluted with sterile demineralised water to a concentration of 10^5^ spores/mL and stored at 4 °C no longer than 3 weeks. The spores were counted using a Neubauer improved cell counter (Marienfeld GmbH, Germany).

The antifungal activity was studied using the cell-free supernatant obtained at the end of the fermentations performed with *P. aeruginosa* #112, the crude RL mixture, and the purified mono-RLs and di-RLs. The assays were performed in Petri dishes (55 mm diameter) containing MEA medium. For the crude RL mixture and the purified mono-RLs and di-RLs, the freeze-dried products were added to the culture medium at different concentrations. When the cell-free supernatant was evaluated, it was used to dissolve the components of the MEA medium instead demineralised water. In all the cases, the pH of the culture medium was adjusted to 5.5. All the media were autoclaved at 121 °C for 15 min. The agar plates were inoculated with 10 µL of the corresponding spore suspension in the centre of the plate. Subsequently, the plates were incubated at 25 °C for 5 days. The fungal growth was determined by measuring the diameter of the growth zone. The percentage of growth inhibition relative to the control was calculated as follows:

1 $$Growth\,inhibition\,x\,( \% )=(1-\,\frac{diameter\,x\,}{diameter\,c\,})\,\times 100$$

where *diameter x* (cm) represents the diameter of the mycelial growth in the medium with the treatment *x*, and *diameter c* represents the diameter of the fungal growth in the corresponding control. All the experiments were performed in triplicate.

### Micelles size measurement by dynamic light scattering (DLS)

The size distribution and the polydispersity indexes (PDI) of the assembled structures formed by the RLs under study (crude RL mixture and purified mono-RLs and di-RLs) dissolved in ultrapure water at different concentrations were measured by DLS using a Malvern Zetasizer Nano-ZS (Malvern Instruments Ltd., UK) at 25 °C. The refractive index of the samples was found to be 1.330, and the scattering angle was 173°. The PDI qualifies the particle size distribution, which here ranged from 0 for monodispersed to 1 for entirely heterodispersed samples. Each sample was analyzed ten times, and information about size distribution by intensity was recorded and averaged.

### Confocal scanning laser microscopy

A Confocal Scanning Laser Microscope (Olympus BX61, Model Fluo View 1000) was used to visualize the structures formed by the crude RL mixture and the mono-RL and di-RL congeners in different aqueous solutions using Nile Red (Sigma-Aldrich Co., USA) as fluorescence probe. Nile Red and RL solutions were prepared in ethanol at a concentration of 1 mg/mL; subsequently both solutions were mixed (700 µL of RLs and 1 µL of Nile Red) and incubated at 150 rpm at room temperature for 30 min. After that, the ethanol was evaporated, and the samples were dissolved in 100 µL of demineralised water or 875 mM NaCl. The samples were immediately observed using the laser excitation line at 559 nm in combination with emission filters BA 575–675. The images were acquired using the program FV10 (Version 4.1.1.5, Olympus).

### Data availability

No datasets were generated or analysed during the current study.
